# A human macrophage – hepatocyte co-culture model for comparative studies of infection and replication of *Francisella tularensis* LVS strain and subspecies *holarctica* and *mediasiatica*

**DOI:** 10.1186/s12866-015-0621-3

**Published:** 2016-01-06

**Authors:** Knut Rennert, Peter Otto, Harald Funke, Otmar Huber, Herbert Tomaso, Alexander S. Mosig

**Affiliations:** Institute of Biochemistry II, Jena University Hospital, 07743 Jena, Germany; Institute of Bacterial Infections and Zoonoses (IBIZ), Friedrich-Loeffler-Institute, Federal Research Institute for Animal Health, 07743 Jena, Germany; Molecular Hemostaseology, Jena University Hospital, 07743 Jena, Germany; Center for Sepsis Control and Care, Jena University Hospital, Jena, 07747 Germany

**Keywords:** Francisella tularensis, Human macrophage, Hepatocyte, Co-culture, Liver

## Abstract

**Background:**

*Francisella tularensis*, a gram-negative bacterium replicates intracellularly within macrophages and efficiently evades the innate immune response. It is able to infect and replicate within Kupffer cells, specialized tissue macrophages of the liver, and to modulate the immune response upon infection to its own advantage. Studies on *Francisella tularensis* liver infection were mostly performed in animal models and difficult to extrapolate to the human situation, since human infections and clinical observations are rare.

**Results:**

Using a human co-culture model of macrophages and hepatocytes we investigated the course of infection of three *Francisella tularensis* strains (subspecies *holarctica* – wildtype and live vaccine strain, and *mediasiatica* - wildtype) and analyzed the immune response triggered upon infection. We observed that hepatocytes support the intracellular replication of *Franciscella* species in macrophages accompanied by a specific immune response inducing TNFα, IL-1β, IL-6 and fractalkine (CX3CL1) secretion and the induction of apoptosis.

**Conclusions:**

We could demonstrate that this human macrophage / hepatocyte co-culture model reflects strain-specific virulence of *Francisella tularensis*. We developed a suitable tool for more detailed in vitro studies on the immune response upon liver cell infection by *F. tularensis*.

**Electronic supplementary material:**

The online version of this article (doi:10.1186/s12866-015-0621-3) contains supplementary material, which is available to authorized users.

## Background

*Francisella tularensis* (*F. tularensis*) is a gram-negative bacterium that causes the zoonosis tularemia [1]. Due to its high infectivity it is considered as a class A bioweapon agent [2]. *F. tularensis* primarily infects and persists in macrophages and is thus used as model bacterium to study adopted strategies to evade primary immune detection [3]. As human infections with *F. tularensis* and related human clinical trials are rare, most of the data available on mechanisms of *F. tularensis* replication are derived from mouse models [4]. However, due to interspecies differences in the immune response and opposing evolutionary strategies for resistance vs. tolerance or resilience to infection [5, 6] it remains difficult to extrapolate results obtained in animal models to the human situation [7].

Currently, four subspecies (ssp.) of *F. tularensis* are generally accepted: *ssp. tularensis*, *holarctica*, *mediasiatica*, and *novicida. F. tularensis* ssp*. tularensis* is highly virulent in hares and the cause of Type A tularemia, whereas *F. tularensis* ssp*. holarctica* is less virulent and causes Type B tularemia; *F. tularensis* subsp. *mediasiatica* is a rare pathogen with unique biochemical characteristics that has only been isolated in Kazakhstan and Turkmenistan in Central Asia and exhibits virulence in hares similar to Type B organisms [8]. Since the infectious dose of *F. tularensis* wild type strains is very low, the attenuated type B live vaccine strain (LVS) thus has often been used as surrogate for the virulent strains. The LVS strain is able to induce diseases in mice similar to those seen in humans, but possesses practically no risk for laboratory personnel [9–11]. Both, virulent and attenuated strains of *F. tularensis,* survive macrophage phagocytosis. They escape into the cytoplasm by preventing acidification and maturation of the phagosome [12–14].

Kupffer cells represent specialized tissue macrophages within the liver [15] and account for 80 – 90 % of the total macrophage pool of the body [16]. In response to *F. tularensis* infection, several morphological alterations of the liver tissue have been observed [17]. In this context, it has been supposed that hepatocytes as well as dendritic cells may support the intracellular replication of *F. tularensis* without undergoing pyroptosis or apoptosis. Moreover, *F. tularensis* is assumed to delay induction of cell death in host cells to its own advantage until exit from its intracellular environment [2].

So far, there is only little knowledge about the infection and replication cycles of *F. tularensis* within human liver tissue. To investigate these processes *in vitro*, we co-cultured primary human monocyte-derived macrophages with the recently developed hepatocyte cell line HepaRG, which differentiates into cells with a hepatocyte phenotype and into cells exhibiting a biliary epithelial cell phenotype [18]. In contrast to other hepatic cells lines, i.e. HepG2 or Hep2/C3A, HepaRG cells remain functionally stable during prolonged culture, self-organize with functional bile canaliculi-like structures and respond to inflammatory cytokines [18]. Using this human macrophage / hepatocyte co-culture approach we characterized the infection and replication of *F. tularensis* ssp. *holarctica*, *spp. mediasiatica* and the attenuated LVS strain.

## Results

**Fig. 1:**
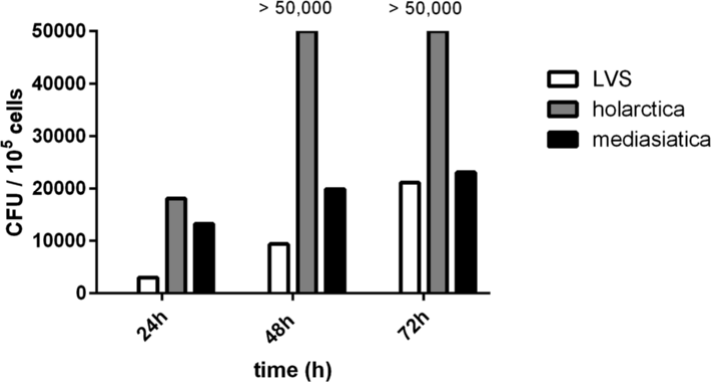
Quantification of colony forming units from cell lysates of macrophage (6 %)/hepatocyte (94 %) co-cultures 24, 48 and 72 h after infection with F. tularensis LVS, spp. holarctica or spp. mediasiatica plated on cysteine heart agar dishes. Data of one representative experiment out of a series of 5 independent experiments for each time point and F. tularensis strain is shown

To characterize the impact of hepatocytes on the intracellular replication rate of *F. tularensis* in mono-cell cultures of macrophages or hepatocytes as well as in macrophage/hepatocyte co-cultures, cells were infected with *F. tularensis* and cultured up to 72 h. The *F. tularensis* replication rate was measured by flow cytometry using an anti- *F. tularensis* lipopolysaccharide (LPS) antibody to detect intracellular bacteria. Macrophages were discriminated from hepatocytes in the flow cytometric analyses by combined gating of FSC/SSC scattering and fluorescence measurement of FITC-labeled antibody directed against the leukocyte marker protein CD45. *F. tularensis ssp. holarctica* and LVS strain were reliably detected in macrophages and hepatocytes. Although it has been concluded from previous studies that all three *F. tularensis* ssp. possess a common LPS lipid A structure [19], we were unable to detect intracellular LPS after infection with *F. tularensis ssp. mediasiatica*. However, presence and replication of intracellular viable bacteria in the hepatocyte / macrophages co-culture of all three *F. tularensis* strains were confirmed by colony forming unit (CFU) assays from lysates of infected cells (Fig. 1, Additional file 1: Figure S1).

**Fig. 2:**
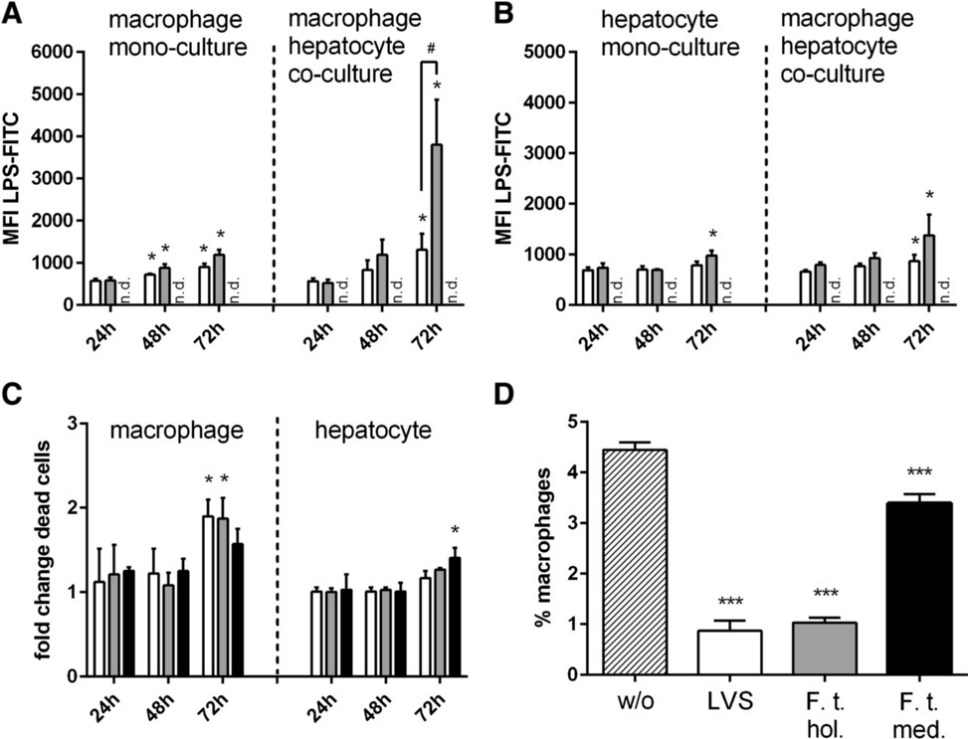
Detection of intracellular LPS and cell death in macrophage (6 %) / hepatocyte (94 %) co-cultures infected with LVS (open bars), *spp. holarctica* (grey filled bars) or *spp. mediasiatica* (black filled bars) and untreated control (hatched bars). **a**-**b** Flow cytometric (FACS) detection of intracellular Francisella tularensis by detection of LPS in **a** macrophages and **b** hepatocytes in the mono-cell culture (left side of dashed line) and co-culture (right side of dashed line) (MFI mean fluorescence intensity); **c** fold change of increase in cell death in macrophages (left side of dashed line) and hepatocytes (right side of dashed line) compared to untreated control in the co-culture; **d** percentage of detectable macrophages in the co-culture 72 h after infection without (w/o) and with incubation of LVS strain (LVS), *F. tularensis holarctica* (F. t. hol.) or *F. tularensis mediasiatica* (F. t. med.). Statistical significance was calculated using student’s *t*-test (**a**-**d**: * *p* < 0.05 compared to corresponding condition 24 h after infection with the respective strain; # *p* < 0.05, statistical significance between indicated conditions calculated with student’s *t*-test)

Replication of *F. tularensis* LVS strain and *F. tularensis ssp. holarctica* was confirmed in monocultures of macrophages and in hepatocytes (Fig. 2a and b, left part) as well as in the co-culture of both cell types (Fig. 2a and b, right part). Interestingly, the highest LPS content was detected in co-cultures with the lowest macrophage content of 6 % in the co-culture (94 % hepatocytes) (Additional file 2: Figure S2A). An increase in the macrophage content in the cell cultures resulted in a diminished LPS signal reflecting first, *F. tularensis* preferentially enter macrophages compared to hepatocytes, and second, in consequence bacterial MOI per individual macrophage cell is reduced in the co-culture.

The strong increase in replication rate of *F. tularensis ssp. holarctica* in macrophages and hepatocytes in the co-culture compared with respective mono-cell cultures, indicates a supportive function of hepatocytes for bacterial replication (Fig. 2a, b). Furthermore, we observed an increased proportion of dead macrophages infected with the LVS strain and *F. tularensis ssp. holarctica* in the co-culture after 72 h, whereas at the same time point *F. tularensis ssp. mediasiatica* induced a significant onset of cell death preferentially in hepatocytes, but not in macrophages (Fig. 2c). An analysis of the total percentage of macrophages revealed that infection with all three *F. tularensis* strains resulted in a significantly diminished macrophage numbers after 72 h in the co-culture. A comparative analysis with different proportions of macrophages in the co-culture revealed that a decline of present macrophages correlated with the detected LPS signal confirming bacterial infection and related subsequent cell death (Additional file 2: Figure S2B). Thus, in the subsequent experiments co-cultures of 6 % macrophage and 94 % hepatocytes were used to enable sufficient bacterial infection rate and allowing an efficient study of the related cellular response. Under these conditions the observed loss of detectable macrophages in response to bacterial infection was most prominent in cell cultures infected with LVS and *F. tularensis ssp. holarctica* compared to *F. tularensis ssp. mediasiatica* (Fig. 2d).

**Fig. 3:**
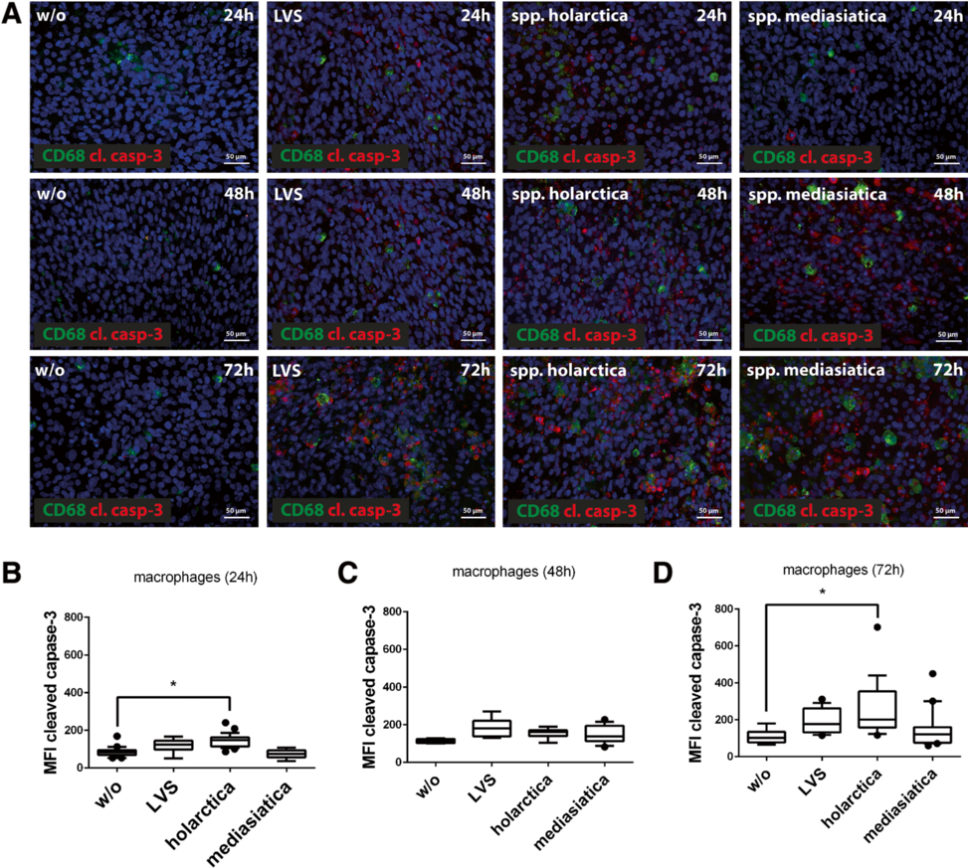
**a** Immunofluorescence staining of macrophages marker protein CD68 (green) and apoptosis marker cleaved caspase-3 (cl. casp-3, red) in co-culture of 6 % macrophages and 94 % hepatocytes. Nuclei are stained with DAPI (blue). Cells were infected with LVS, *spp. holarctica* and *spp. mediasiatica* and subsequently co-cultured for 24, 48 and 72 h, respectively. **b-d** Quantification of the mean fluorescence intensity (MFI) of labeled antibody for detection of the apoptosis marker cleaved caspase-3 (cCasp-3) in macrophages b) 24h, c) 48h and d) 72h post infection. Whisker dot plots show boxes ranging from 10th (lower box boundary) to 90th (upper box boundary). Vertical line inside the box marks the median. Outliers are shown as filled circles. Statistical significance was calculated using one-way ANOVA with Dunnett's multiple comparisons (* *p* < 0.05 for indicated conditions)

Induction of apoptosis in macrophages in response to infection with all three *F. tularensis* strains was detected in the co-cultures with hepatocytes at the level of cleaved caspase-3. In these assays, infection with *F. tularensis ssp. holarctica* caused the highest rate of apoptosis induction in macrophages 24 h and 72 h post infection (Fig. 3b-d, b).

It has been demonstrated in mice that after 16 h of intravenous infection with *F. tularensis* infectious foci in liver tissue are formed and accompanied by infiltration of both neutrophils and monocytes [20]. Infected mice developed typical signs of hepatitis within 24–72 h post infection [21] and during this period, pro-inflammatory cytokines, including interleukin (IL)-1β, IL-6, and tumor necrosis factor (TNF) α were secreted at the active sites of infection [11, 22–24]. To characterize the inflammatory response in our humanized co-culture model the secretion of these pro-inflammatory cytokines in response to *F. tularensis* infection was measured in the supernatant of the cell culture by cytometric bead arrays (CBA). In addition, the release of fractalkine (FKN, CX3CL1), a chemokine that controls the survival of invading monocytes and their differentiation into functionally diverse macrophage subsets upon liver injury [25] was analyzed by CBA. In chronic liver injury FKN elicits liver protective functions via its receptor CX3CR1 by promoting hepatic macrophage survival and restriction of pro-inflammatory macrophage polarization [26, 27].

**Fig. 4:**
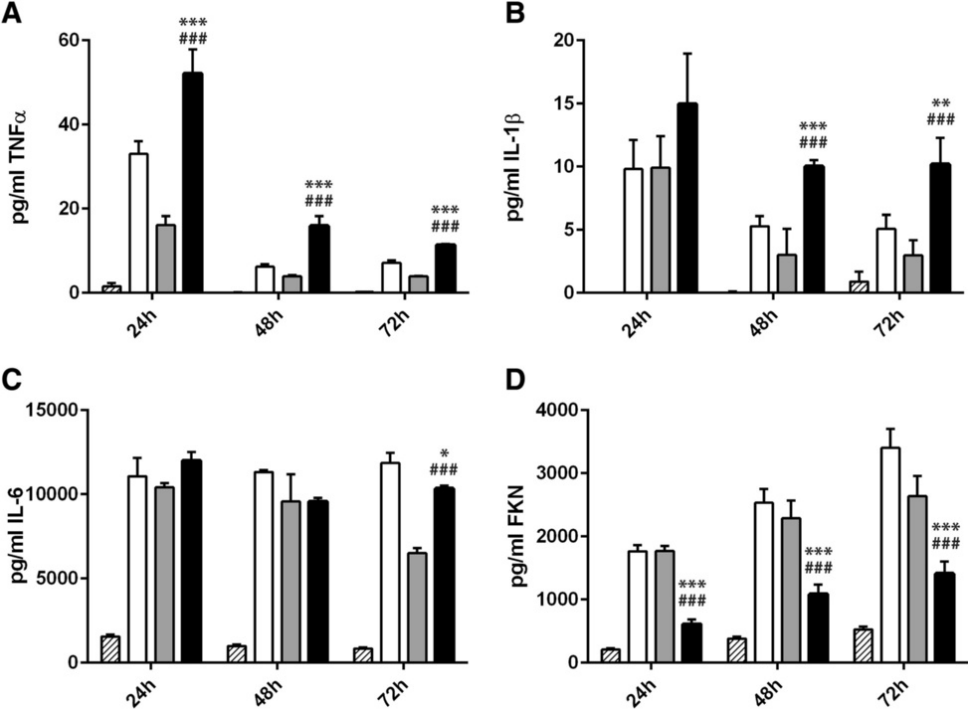
Cytokine secretion by macrophage/hepatocyte co-cultures of **a** TNFα, **b** IL-1β, **c** IL-6 and **d** fractalkine (FKN) in response to infection with LVS (open bars), *spp. holarctica* (grey filled bars) or *spp. mediasiatica* (black filled bars) or untreated control (hatched bars) after 24, 48 and 72 h of cell culture. Statistical significance was calculated using student’s *t*-test: *** *p* < 0.001 compared to identical time point after infection with LVS; ### *p* < 0.001 compared to identical time point after infection with *spp. holarctica*

We observed a robust and sustained inflammatory response upon infection with all three *F. tularensis* strains up to 72 h after infection. TNFα and IL-1β levels were highest 24 h after infection and subsequently declined in the further course of culture (Fig. 4a, b). A release of IL-6 was similarly triggered by infection with all *Francisella* strains after 24 h. Whereas IL-6 levels declined in *F. tularensis ssp. holarctica* and *ssp. mediasiatica* infected co-cultures up to 72 h, IL-6 levels remained stable upon infection with the LVS strain (Fig. 4c). In contrast to the release of pro-inflammatory cytokines TNFα, IL-1β and IL-6, the release of FKN increased from 24 h up to 72 h post infection (Fig. 4d). Interestingly, FKN release negatively correlated with pro-inflammatory cytokine release in a time-dependent as well as strain-specific manner, indicating that the release of the FKN chemokine negatively correlates with *F. tularensis*-mediated inflammation response. Among all three *F. tularensis* strains tested, *F. tularensis ssp. mediasiatica* infection induced the secretion of only low FKN levels but high amounts of TNFα and IL-1β were secreted relative to the LVS strain and *F. tularensis ssp. holarctica*.

**Fig. 5:**
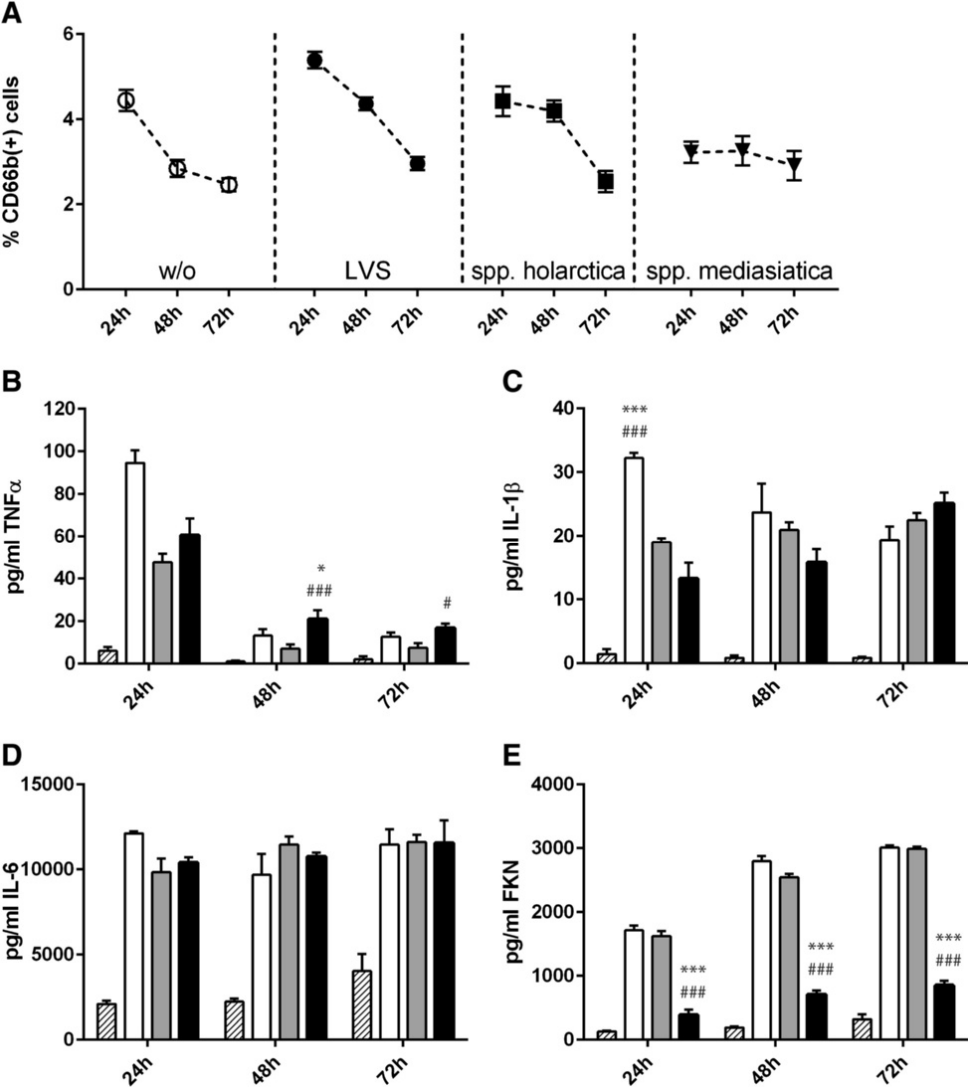
White blood cell (WBC) migration and cytokine secretion by macrophage (6 %) / hepatocyte (94 %) cultures co-incubated with 2 x10^5^ WBC in a transwell filter insert. **a** Percentage of detected CD68b positive granulocytes by flow cytometry 24, 48 and 72 h post infection. Cytokine secretion of **b** TNFα, **c** IL-1β, **d** IL-6 and **e** fractalkine (FKN) in response to infection with LVS (open bars), *spp. holarctica* (grey filled bars) or *spp. mediasiatica* (black filled bars) or untreated control (hatched bars) after 24, 48 and 72 h of cell culture. Statistical significance was calculated using student’s *t*-test: * *p* < 0.05, *** *p* < 0.001 compared to identical time point after infection with LVS; # *p* < 0,05, ### *p* < 0.001 compared to identical time point after infection with *spp. holarctica*

*F. tularensis* attempts to evade the primary immune response by intracellular replication [3]. To study whether its presence in the cell culture is masked to suspended immune cells, we co-incubated infected cells with human white blood cells (WBC) seeded in a transwell filter above the co-cultures. In this setting we were not able to detect a significant number of migrating and adhesive leukocytes to the infected cell cultures. Only small amounts of less than 5 % migrating granulocytes were detectable (Fig. 5a) in naïve and infected cell cultures, but without significant differences in respect to cell numbers (Fig. 5a). Although secretion of TNFα and IL-1β was increased, we found only minor alterations of the time- or strain-dependent IL-6- and FKN-release (Fig. 5b-e) in presence of co-incubated WBC.

## Discussion

It has been suggested that hepatocytes as well as dendritic cells support the intracellular replication of *F. tularensis* without undergoing pyroptosis or apoptosis [2]. We could confirm this concept derived from the mouse models in the human cell culture model for the virulent subspecies *F. tularensis mediasiatica* but not for the subspecies *holarctica* or the attenuated LVS strain. In the human co-culture model we could detect a significant loss of macrophages infected with *F. tularensis ssp. holarctica* and the LVS strain, whereas the percentage of viable macrophages only slightly decreased after infection with *F. tularensis ssp. mediasiatica*.

*F. tularensis* is presumed to delay induction of cell death in host cells to its own advantage until exit from its intracellular environment [2]. *F. tularensis ssp. mediasiatica* was found to efficiently adopt this strategy by preventing macrophage cell death in order to allow efficient replication rates. It was shown that infection of the macrophage-like J774 cell line with LVS results in apoptosis [28]. However, the number of bacterial burden was not affected. In this context, a role of the caspase-3-mediated cell death in favoring bacterial dissemination was assumed, but this mechanism is restricted to tissues in which macrophages play a central role in pathogen uptake and killing, such as the liver [2]. In mice, death of animals infected with *F. tularensis* appears to result from widespread sepsis and inflammation [29–31], and mortality was correlated with the extent of the inflammatory response [17] including release of pro-inflammatory IL-6, an early diagnostic marker of bacterial sepsis [32].

We observed the strongest pro-inflammatory response upon infection with *F. tularensis ssp. mediasiatica* that also induced the highest rate of apoptosis in the co-culture model of all tested *F. tularensis* strains. We further observed a reduced level of FKN, a chemokine released by cleavage from hepatocytes upon inflammation [33] and that is able to counteract liver inflammation [26] in response to *F. tularensis ssp. mediasiatic*a infection. In mice, lethal infection with *F. tularensis* is associated with hypercytokinemia and biochemical markers for sepsis [29–31]. This sepsis is assumed to result from delayed cytokine up-regulation and insufficient recruitment of inflammatory cells [31]. Although we observed a slight increase of TNFα and IL-1β secretion upon WBC co-incubation, no effects on IL-6 release were observed. Based on the diminished immune response of co-incubated WBC we conclude that intracellular bacteria are mainly hidden from WBC, which impedes an efficient bacterial clearance.

On one hand, the observed high rate of apoptosis induction in macrophages infected with *F. tularensis* provides the host with the advantage of early elimination of infected cells and removal of microbial replication niches. On the other hand, the extensive cell death of tissue macrophages, which is a hallmark of virulent *F. tularensis* infection [2], and the reduced recruitment of phagocytes to the sites of infection favors the dissemination of bacteria and diminishes important innate immune responses [2, 29]. This double-edged sword needs to be tightly regulated by the host to ensure an efficient host defense against *F. tularensis* infection.

## Conclusions

We could demonstrate that *F. tularensis* infection and related cellular immune response can be investigated in a human cell co-culture model of monocyte-derived macrophages and hepatocytes. This co-culture model depicts strain-specific virulence and the associated pathogenic potential of the tested *F. tularensis* strains. Using this model we could demonstrate that *F. tularensis* strains efficiently hide and replicate in macrophages depending on their virulent potential and thereby avoid detection by co-incubated WBC and subsequent aggravated immune response. We believe that the cell co-culture approach presented herein is a valuable tool for further, more detailed *in vitro* studies of *F. tularensis* pathophysiology. The potential of this co-culture models to study other intracellular persisting pathogens affecting the human liver will be characterized in further studies.

## Methods

### Cell isolation and culture

#### *HepaRG hepatocytes*

HepaRG cells were obtained from Biopredic International (Rennes, France). Undifferentiated HepaRG were seeded at a density of 2,7 × 10^4^ cells/cm^2^ per well of a 12 well-plate and cultured in William’s Medium E (Biochrom, Berlin, Germany) containing 10 % (v/v) FCS (GIBCO, Darmstadt, Germany), 5 μg/ml insulin (Sigma-Aldrich, Steinheim, Germany), 2 mM glutamine (GIBCO), 5×10^−5^ M hydrocortisone-hemisuccinate (Sigma-Aldrich) and 100 U/ml penicillin/100 μg/ml streptomycin mixture (Pen/Strep) (GIBCO). The cells were cultured in a humidified cell incubator at 5 % CO_2_ and 37 °C for 14 days before differentiation. Medium was renewed every 3–4 days. Cell differentiation was induced for 14 days in the presence of 2 % (v/v) DMSO (Sigma-Aldrich) as described [34]. At day 8 before starting the infection with bacteria. *Primary macrophages:* PBMC were isolated from whole blood which was collected from healthy human donors that were informed about the aim of the study and gave written informed consent. The study was approved by the ethics committee of the Jena University Hospital. PBMCs were isolated by Ficoll density gradient centrifugation as described previously [35, 36] and monocytes isolated from PBMCs with the Dynabeads ® CD14 Isolation Kit according to the manufacturer’s protocol (Life Technologies, Darmstadt, Germany). For macrophages differentiation monocytes were seeded into each well of 12 well plates and cultured for 6 days in X-VIVO 15 medium (Lonza, Cologne, Germany) supplemented with 10 % (v/v) autologous human serum, 10 ng/ml human granulocyte macrophage colony-stimulating factor (GM-CSF) (PeproTech, Hamburg, Germany) without antibiotics. Medium was exchanged after three days. For co-culture of macrophages and hepatocytes, monocytes were plated and differentiated for 6 days to macrophages. HepaRG cells were subsequently added to the macrophage culture. Total percentage of macrophages in the co-culture was controlled by seeding different monocyte cell densities and filling up with hepatocytes to a resulting total cell density of 1.0 × 10^5^ cell/cm^2^ used for all tested co-cultures.

#### White blood cell isolation

WBCs were isolated from 9 ml EDTA-blood using the erythrocyte lysis buffer of the QIAamp RNA Blood Mini Kit (Qiagen) followed by three washing steps in PBS containing 2 mM EDTA.

### Francisella tularensis culture

*Francisella tularensis ssp. holarctica* (FLI isolate 06 T-0001 and live vaccine strain-LVS) *and ssp. mediasiatica* (F63) were provided by the cryobank of the German National Reference Laboratory for Tularemia, plated and cultured on cysteine heart agar (CHA) in a humidified incubator at 4,5 % CO_2_ and 37 °C for 72 h. Subsequently, colonies were suspended in PBS.

### Infection

At the day of infection, bacterial suspensions were adjusted to an OD_600_ = 0.4, corresponding to 5 × 10^9^ bacteria/ml. Cell cultures were incubated with *F. tularensis* bacteria (MOI 100:1) for 2 h in a humidified cell incubator at 4,5 % CO_2_ and 37 °C. Subsequently, the bacterial suspension was removed, washed with Williams medium E and further incubated in William’s Medium E containing 10 % (v/v) FCS, 5 μg/ml insulin, 2 mM glutamine, 5×10^−5^ M hydrocortisone-hemisuccinate and 10 μg/ml gentamicin (Sigma-Aldrich, Taufkirchen, Germany) for 30 min to kill extracellular bacteria. Infected cells with persisting *Francisellae* were than washed with PBS and cultured for indicated times in William’s Medium E containing 10 % (v/v) FCS, 5 μg/ml insulin, 2 mM glutamine, 5×10^−5^ M hydrocortisone-hemisuccinate without antibiotics.

### White blood cell co-incubation

2 × 10^5^ freshly isolated white blood cells were transferred into each cell culture inserts with 8 μm pores (Merck Millipore, Darmstadt) hanging above *F.tularensis* pre-inoculated co-cultures of HepaRG/macrophages. WBCs were incubated for up to 72 h with the infected co-cultures. Subsequently, inserts were discarded and cells in the subjacent well were harvested for flow cytometry. Granulocytes and T-cells were stained with fluorochrome-conjugated antibodies against CD66b and CD3 (BD Biosciences) and analyzed by flow cytometry.

### Immunofluorescence staining

Cells were fixed with 4 % paraformaldehyde for 10 min at room temperature (RT). Staining was done with antibodies against CD68 (BD Biosciences, Heidelberg, Germany), cleaved caspase 3 (cCasp-3) (Cell Signaling Technology, Leiden, Netherlands), and goat-anti-rabbit-Cy3 (Dianova, Hamburg, Germany) and goat-anti-mouse-AlexaFluor488 (AF488) as secondary antibodies, and DAPI (Life Technologies, Karlruhe, Germany). Samples were embedded into fluorescent mounting medium (Dako, Hamburg, Germany). Subsequently, imaging was performed on an AXIO Observer Z1 fluorescence microscope equipped with Apotome 2 (Carl Zeiss AG, Jena, Germany).

### Image analysis

Analysis of cleaved caspase-3 fluorescence signals in macrophages was done with ImageJ2 software. Specifically, macrophages were detected based on the expression of the cell-type-specific marker protein CD68 stained with AF488 coupled secondary antibody and cell borders of the cell marked as region of interest (ROI). ROI’s were than analyzed for fluorescence signals of cCasp-3 and mean fluorescence intensity (MFI) was calculated and plotted in a whisker box plot diagram.

### Colony forming unit assay

After indicated incubation times, cells were lysed in 1 ml Aqua bidest. for 10 min at RT. Subsequently, 20–100 μl of the lysate were transferred and streaked on cysteine heart agar without antibiotics. Agar plates were cultivated for 2 days in a humidified incubator at 4,5 % CO_2_ and 37 °C. Afterwards colonies were counted.

### Live/dead staining

Macrophages and hepatocytes were detached from cell culture dishes using 4 mg/ml Lidocaine and 5 mM EDTA (Sigma Aldrich) in PBS (Lonza,, Cologne, Germany), centrifuged at 300 x g at room temperature (RT) for 6 min, washed two times with PBS at RT and stained with LIVE/DEAD® Cell Viability assay (Life Technologies) for 30 min according to the manufacturer’s instructions.

### FACS analysis

Macrophages were stained with antibody against CD45-APC-Cy7 (BD Bioscience, Heidelberg, Germany) and fixed with Inside Stain kit (Miltenyi Biotec, Bergisch Gladbach) according to manufacturer’s recommendations for permeabilization of cells thus allowing detection of *F. tularensis* LPS. Intracellular *Francisellae* were stained with 2,5 μg/ml FITC-conjugated antibody versus *F. tularensis* LPS (clone FB11 - HyTEST Ltd., Turku, Finland). Clone FB 11 does not recognize LPS of *F. tularensis ssp. mediasiatica.* Flow cytometry was performed on a BD FACS-Canto II (BD Biosciences) with FACSDiva software and analyzed using FlowJo X software (FlowJo LLC, Ashland, OR, USA).

### Cytometric bead array (CBA)

Supernatants were collected after indicated time periods and immediately frozen at −80 °C. Cytokines were detected using CBA assay (BD Biosciences) according to the manufacturer’s protocol. Secretion of TNFα, IL-1β, IL-6, fractalkine and IFNα was analyzed using standard CBA flex sets. Analysis was performed on a BD FACS-Canto II cytometer with FACSDiva software. Data analysis was performed using FCAP Array V3 software (Softflow, Pecs, Hungary).

### Statistics

All results are represented as mean of the performed experiments with standard deviation. Statistic tests were done with two-tailed, non-paired Student’s *t*-test or one-way ANOVA with Dunnett’s multiple comparisons test between indicated conditions. Statistical analysis was performed using GraphPad Prism 6.07 software (Graphpad Software, La Jolla, CA, USA). For CFU analysis data of one representative experiment out of a series of 5 independent experiments for each time point and F. tularensis strain is shown in Fig. 1 and Additional file 1: Figure S1.

## Additional files

Additional file 1: Figure S1. Colony forming units assay from cell lysates of macrophage (6 %)/hepatocyte (94 %) co-cultures 24, 48 and 72 h after infection with *F. tularensis* LVS, *spp. holarctica* or *spp. mediasiatica* plated on cysteine heart agar dishes. Data of one representative experiment out of a series of 5 independent experiments for each time point and F. tularensis strain is shown. (TIF 77516 kb) Additional file 2: Figure S2. Detection of intracellular LPS in macrophage / hepatocyte co-cultures infected with LVS (open bars), *spp. holarctica* (grey filled bars) or *spp. mediasiatica* (black filled bars) and untreated control (hatched bars). A) Different amounts of macrophages in the co-culture were tested (6, 12 and 22 % of macrophages on total cell count). Flow cytometric detection of intracellular LPS in macrophages (MFI mean fluorescence intensity); B-D) percentage of remaining detectable macrophages after infection of the co-cultures with B) 6 % macrophages/94 % hepatocytes, C) 12 % macrophages/ 88 % hepatocytes and D) 22 % macrophages/ 88 % hepatocytes 72 h post infection. (TIF 32735 kb)

## Abbreviations

CHA: Cysteine heart agar
CFU: Colony forming unit
CX3CL1: Fractalkine
DMEM: Dulbecco’s modified eagle medium
ECM: Endothelial cell medium
FKN: Fractalkine
*F. tularensis*: *Francisella tularensis*
GM-CSF: Granulocyte-macrophage colony stimulating factor
IL: Interleukin
LPS: Lipopolysaccharide
LVS: Live vaccine strain
MOI: Multiplicity of infection
Pen/Strp.: Penicillin/Streptomycin
RT: Room temperature
spp.: Subspecies
WBC: White blood cell
v/v: Volume/Volume
