# Supplementary figures and images for: A human macrophage – hepatocyte co-culture model for comparative studies of infection and replication of Francisella tularensis LVS strain and subspecies holarctica and mediasiatica
Source: BMC Microbiol. 2016 Jan 6;16:2. doi: 10.1186/s12866-015-0621-3 (PMC4704405; doi:10.1186/s12866-015-0621-3)

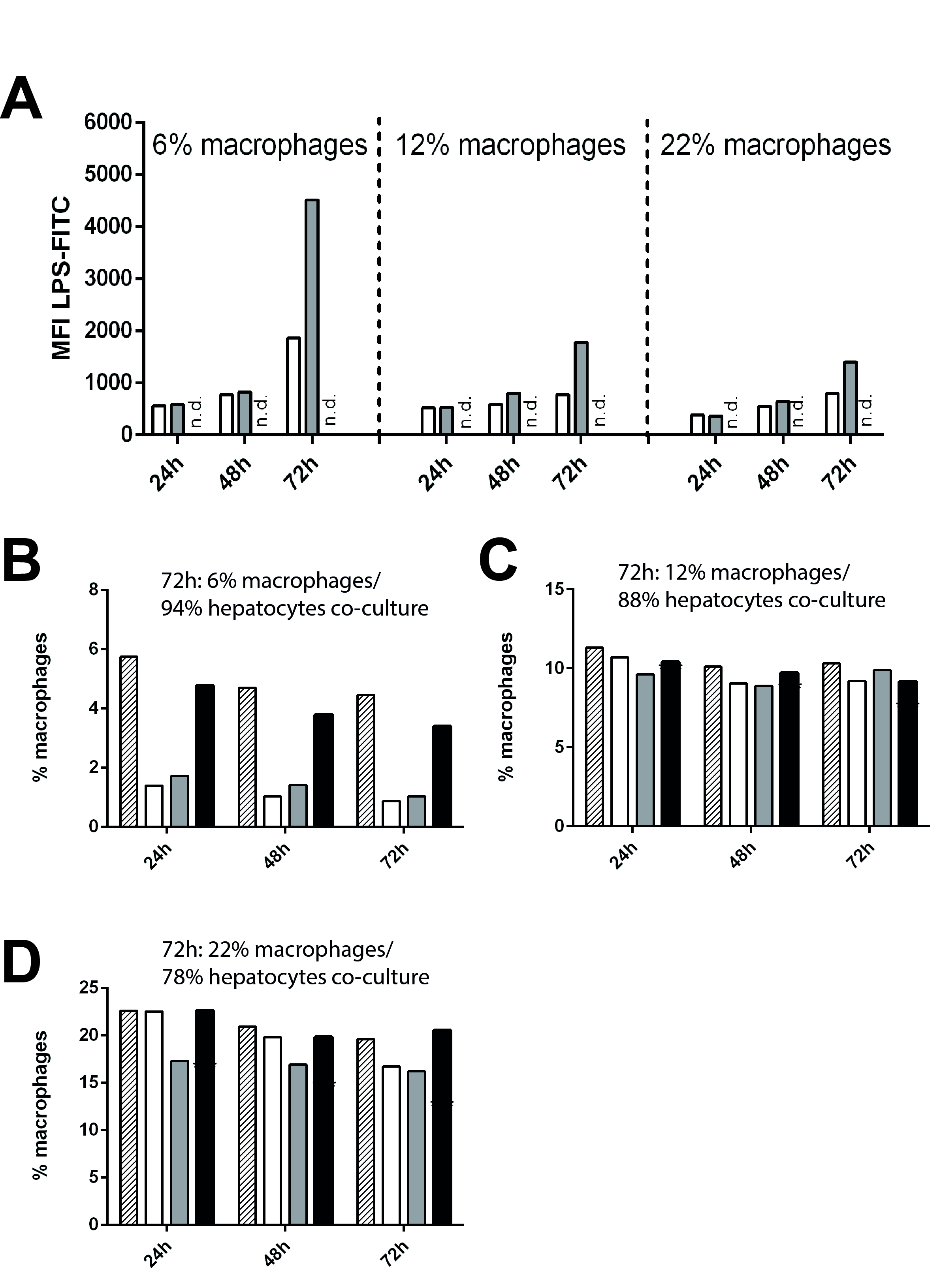

Supplement: Additional file 2: Figure S2. — Detection of intracellular LPS in macrophage / hepatocyte co-cultures infected with LVS (open bars), spp. holarctica (grey filled bars) or spp. mediasiatica (black filled bars) and untreated control (hatched bars). A) Different amounts of macrophages in the co-culture were tested (6, 12 and 22 % of macrophages on total cell count). Flow cytometric detection of intracellular LPS in macrophages (MFI mean fluorescence intensity); B-D) percentage of remaining detectable macrophages after infection of the co-cultures with B) 6 % macrophages/94 % hepatocytes, C) 12 % macrophages/ 88 % hepatocytes and D) 22 % macrophages/ 88 % hepatocytes 72 h post infection. (TIF 32735 kb) [file 12866_2015_621_MOESM2_ESM.tif]
